# Human deforestation outweighs future climate change impacts of sedimentation on coral reefs

**DOI:** 10.1038/ncomms2986

**Published:** 2013-06-04

**Authors:** Joseph Maina, Hans de Moel, Jens Zinke, Joshua Madin, Tim McClanahan, Jan E. Vermaat

**Affiliations:** 1 Department of Biological Sciences, Macquarie University, Sydney, New South Wales 2109, Australia; 2 Marine Programs, Wildlife Conservation Society, Bronx, New York 10460, USA; 3 Faculty of Earth and Life Sciences, Institute of Environmental Studies, VU University Amsterdam, Amsterdam, The Netherlands; 4 School of Earth and Environment, Australian Institute of Marine Science, UWA Oceans Institute, University of Western Australia, Perth, Western Australia, Australia; 5 Faculty of Earth and Life Sciences, Section of Earth Sciences and Economics, VU University Amsterdam, Amsterdam, The Netherlands

## Abstract

Near-shore coral reef systems are experiencing increased sediment supply due to conversion of forests to other land uses. Counteracting increased sediment loads requires an understanding of the relationship between forest cover and sediment supply, and how this relationship might change in the future. Here we study this relationship by simulating river flow and sediment supply in four watersheds that are adjacent to Madagascar’s major coral reef ecosystems for a range of future climate change projections and land-use change scenarios. We show that by 2090, all four watersheds are predicted to experience temperature increases and/or precipitation declines that, when combined, result in decreases in river flow and sediment load. However, these climate change-driven declines are outweighed by the impact of deforestation. Consequently, our analyses suggest that regional land-use management is more important than mediating climate change for influencing sedimentation of Malagasy coral reefs.

## Abstract (web summary)

Forest cover up-river influences the sediments reaching coral reefs, but how this relationship is affected by future climate change is not clear. In a study of the Malagasy coral reefs, Maina *et al.* find that regional land-use management is more important than mediating climate change for reducing reef sedimentation.

## Graphical abstract

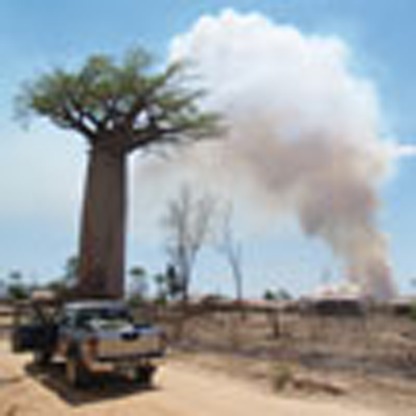

Madagascar is a global biodiversity hotspot and is therefore of high conservation priority^1^,^2^. Biologically rich forests have experienced landscape-level conversion and deforestation primarily from anthropogenic agents, with ~90% loss of original forest cover over the last ~2000 years since human arrival^3^,^4^. Despite attempts at protection that date back two centuries and investment of hundreds of millions of dollars, forest conservation goals appear to remain elusive, and forest destruction continues^1^,^5^. Changes in vegetation cover and soil have caused disruption of water flow and soils in drainage basins’ hydrological cycles, potentially undermining the resilience of physically and biologically linked ecosystems^5^,^6^,^7^. Meanwhile, the conservation of coral reefs in a high-carbon dioxide world in Madagascar and in other tropical countries requires as one of the key strategy outcomes a reduction in the load of terrestrial sediment, nutrients and other pollutants^8^,^9^,^10^. Therefore, quantifying hydrological changes of these coastal catchments under different climate and land use-land cover change (LULCC) scenarios will provide a basis for understanding and predicting the influence of land-use on sedimentation of near-shore marine areas, and subsequently help to identify land-use practices that will reduce the impacts^11^.

Pronounced climate change and LULCC represent the two primary challenges that most ecosystems will face this century^12^. These changes will directly affect the hydrology of the land surface through changes in evapotranspiration and ground water^13^,^14^ and the amount of sediment and fresh water discharged into marine coastal zones^6^. Predicting the impact of climate change and LULCC on the spatial and temporal fluvial dynamics in the tropics is complicated because of poor data availability, strong seasonality and high-intensity events such as El Niño-Southern Oscillation^15^,^16^. Therefore, identifying and separating the human impacts on fluvial dynamics relative to background processes and natural variability is challenging.

Using a hydrological and sedimentation modeling framework developed for Madagascar^6^, we compare both the effects of LULCC and projected climate change on river discharge and sediment supply from four Malagasy catchments (the south-west, west, north-west and north-east; Fig. 1). Malagasy watersheds represent socio-ecologically sensitive coastal catchments, and because latitudinal range and island aspect explain a large amount of environmental variability, they capture characteristically different climatic zones, and therefore represent a range of environments found throughout the global tropics^17^. The catchments are adjacent to four main regions supporting Malagasy coral reefs, and the resource use in these watersheds along with fishing are key components of the coral reef social*-*ecological system dynamics in Madagascar and throughout the tropics^18^,^19^. Using our integrated framework, we find that an increase in air temperature and a decline in precipitation will lead to a general decline in river flow and sediment supply to coral reef areas. However, these climate change-driven declines are outweighed by the impact of deforestation. To date, removal of natural forest has increased sediment supply up to five-fold since human settlement. Sediment supply is projected to increase further by 54–64% if 10–50% of natural forest is removed, but could be reduced by 19–68% if 10–50% of natural forest is restored. All in all, we demonstrate an integrated terrestrial–marine framework for informing decisions concerning sediment supply to coral reefs.

## Results

### Effects of changes in temperature and precipitation

To determine the relative influence of climate change on sedimentation, we derived projected multi-model ensemble mean changes in precipitation and temperature from six GCMs, in which three Intergovernmental Panel on Climate Change (IPPC) emission scenarios (Special report on Emission Scenarios, B1, A1b and A2)^20^ were represented (Methods). Temperature is projected to increase by ~1–4 °C in all seasons and in the four regions depending on different projections of three climate scenarios—by the end of the twenty-first century, with SRES B1, A1b and A2 showing small, moderate and large increases, respectively (Fig. 2). The multi-model ensemble predictions indicate a slight increase in precipitation (5–10%) for the wet season and a further drying across the four regions during the dry season (5–10% in the NE and NW) that is especially robust in the subtropical west and southwest (up to 20%; Fig. 2). Generally, projections suggest a tendency for wet seasons to get wetter in climatologically wetter regions (that is, NE), and for dry seasons to get drier in drier regions (that is, SW, W). These patterns are qualitatively in agreement with previous analyses for the south-western mainland of Africa (for example, in the studies of Tadross *et al.*^21^ and Christensen *et al.*^22^), and for regional scale^18^.

### Effects of climate change and LULCC

Climate-mediated changes in precipitation and temperature have variable effects on river flow between regions and scenarios (Fig. 2). The south-western catchment has reduced water balance and the greatest decline in river flow in all emission scenarios (ensemble median 39–56%); NE and NW have moderate decline at 15–30%, while the western catchment had the lowest decline of ~10%. The projected dry and hot climatic conditions will have substantial impacts on ground water resources^13^ and soil moisture^23^ especially for the drier southwest, factors that contribute to the simulated declines in river discharge and sediment yield. This also highlights the vulnerability of water resources in this region, even as sedimentation pressure on coastal ecosystems eases marginally (~10%) by the end of the twenty-first century with the unlikely no-change in LULCC scenario.

To determine the influence of LULCC on sedimentation, we constructed different possible land-use inputs for the hydrological models (Methods). These configurations were based on present-day land-use (2000), undisturbed natural forest based on bio-climate zones^4^,^24^, and spatially explicit island-wide deforestation and afforestation targets of 10, 25, 50 and 100%. The afforestation management regimes assume a sustained environmental campaigns and a heightened role for conservation than that currently exists, while deforestation would be associated with continued loss of forest cover or other changes in policy and action that promote the removal of forests.

The impacts of LULCC on sediment yields demonstrate the profound influence of human actions on tropical hydrology^16^, and the potential of management measures to curb deforestation and to increase forest coverage. On one extreme, afforestation of all areas to natural conditions would decrease sediment yield in all the four regions by 75–80% (Fig. 3; Table 1), while on the other extreme, forest conversion in all areas would increase sediment yield by up to five-fold in the NE and two-fold in the other regions (Fig. 3; Table 1).

Simulated changes in sediment yield when plotted against change in forest cover for each region indicates regionally specific trajectories owing to the bio-climatic differences and the disproportionate effects of forest manipulation policies on forest cover (Fig. 3). Sediment changes are not dependent on the different predictions of the three climate scenarios. In the NE and NW regions, 25% island-wide afforestation scenario is estimated to restore 11% and 25% forest cover respectively, while a 50% scenario would restore 41 and 56%, respectively. These afforestation targets for NE and NW would lead to substantial declines (~60–80%) in the mean annual sediment yield.

The NE is one of the most hydrologically sensitive watersheds, where small changes in forest cover are projected to produce large changes in sediment yield. The NE region is still covered by relatively large areas of low-lying tropical forests, albeit fragmented, as a result of a low population density^4^. The relatively high forest coverage in this region relative to other regions is also consistent with our forest manipulation models (Fig. 3), which predict low susceptibility to deforestation due to the steep terrain and less-developed infrastructure. Sediment yield changes along the forest cover gradient for the SW and W regions depict nearly identical response behavior patterns, perhaps owing to relatively similar climate and natural vegetation of mostly dry forest areas with less soil-binding vegetation^25^. In this region, 25% island-scale forest conversion (removes ~44 and 46% of forest cover in SW and W respectively) leads to up to two-fold increase in sediment yield. It appears that at this level of deforestation, topsoils are completely depleted with sediment yield reaching a saturation point, which does not change with further decreases in forest cover. For the SW, 10 and 25% island-scale afforestation targets would not have much impact on sediment yield, as this increases the forest cover in the watershed by only 4 and 12%, respectively. Significant sediment declines in the SW would be achieved with implementation of 50% island-scale afforestation target, which would add 29% of forested area and consequently reduce sediment yield by ~50%. Similarly in the west, an estimated 60% decline in sediment yield is achieved with an island-scale afforestation target of 50%.

## Discussion

Model results provide insights into the effects of forest conversion in the face of rising temperatures under climate change as well as afforestation policies and goals appropriate for the respective regions. Although climate change may be expected to exacerbate these challenges through forest diebacks^23^,^26^,^27^, forest conversion remains the principal contributor to increased sedimentation of the near-shore marine environments. Future industrial development, such as dredging activities (currently not a concern), could locally exacerbate the effects of forest conversion^28^,^29^. Increased sedimentation and poor water quality compromises resistance of corals to thermal stress and their potential to recover from bleaching events, resulting in a global deterioration of reef structure and ability of these ecosystems to sustain their characteristic complex ecological interactions^30^,^31^. As it is unlikely that rising global average temperature will stabilize at 2 °C above a pre-industrial levels^32^,^33^, it is foreseen that unprecedented loss of species and widespread bleaching is a likely scenario^32^,^34^,^35^. Therefore local-scale mitigation through curbing sediment pollution has been promoted as particularly relevant for many tropical coastal communities who depend directly on marine resources for their livelihood. It is an important realization that the management of land-use offers a practical solution to reducing sedimentation and contributing to the resilience and adaptability of coral reefs facing both direct and indirect threats of rising CO_2_ (ref. 20).

The Malagasy and other governments are experiencing increased interests from the broader donor, environmental and conservation communities to reverse deforestation and maintain the hydrological and biological functions of forests^5^. However, forest conservation in Madagascar faces several challenges and competing interests that create a tension between resource allocation and conservation, such as increasing population, demand for agricultural land, and mineral exploration and mining^36^. Further, there are technical challenges that include monitoring forests dynamics and predicting how forests will respond to future climate change^16^ in order to assess the sustainability and social equity of different management regimes^37^. For instance, forest diebacks due to drought are likely to hamper reforestation and lead to further losses of agricultural productivity and forest cover^23^,^26^,^38^, especially in the drier regions of W and SW. These challenges are monumental but are achievable with broader international support along with the government and community’s good will and engagement. Finally, if afforestation were adopted and applied comprehensively and consistently within a broader management policy and actions framework, it offers promise for sustainable environmental outcomes in the face of climate change in one of the world’s most important biodiversity hotspots.

## Methods

### Models

We used two existing models (STREAM^39^ and N-SPECT^40^ that have been developed for Madagascar^6^ that utilize present-day climate and land-use data (1950–2006) to estimate 57-year time series of river discharge (m^3^ s^−1^) and sediment yield (tonnes per year)^6^. In these calibrated and validated models, we substituted present-day climate and land-use data with future climate projections and LULCC scenarios, in order to simulate river discharge and sediment yield with new climate and land-use data forcing.

We used the STREAM (Spatial Tools for River Basins, Environment and Analysis of Management options)-distributed hydrological model to simulate monthly river flow^35^. STREAM simulates the water balance for each grid using a limited number of parameters, including the spatial-temporal precipitation and temperature trends, elevation, land-cover and soil water storage capacity. STREAM can provide several outputs including, river flow maps for selected *x–y* points in the watershed(s) being simulated, or as spatial maps of river flow per time step. N-SPECT^36^ (Non-Point Source Pollution and Erosion Comparison Tool), developed by National Oceanic and Atmospheric Administration (NOAA), was used to calculate sediment yield per unit area. N-SPECT combines data on elevation, slope, soils, precipitation and land cover to derive estimates of runoff, erosion and pollutant sources (nitrogen, phosphorous and suspended solids), including estimates of sediment and pollutant accumulation in stream and river networks. Erosion rates and sediment loads are calculated using the Revised Universal Soil Loss Equation (RUSLE) and Modified Universal Soil Loss Equation (MUSLE).

### Climate change scenarios

**Figure 1:**
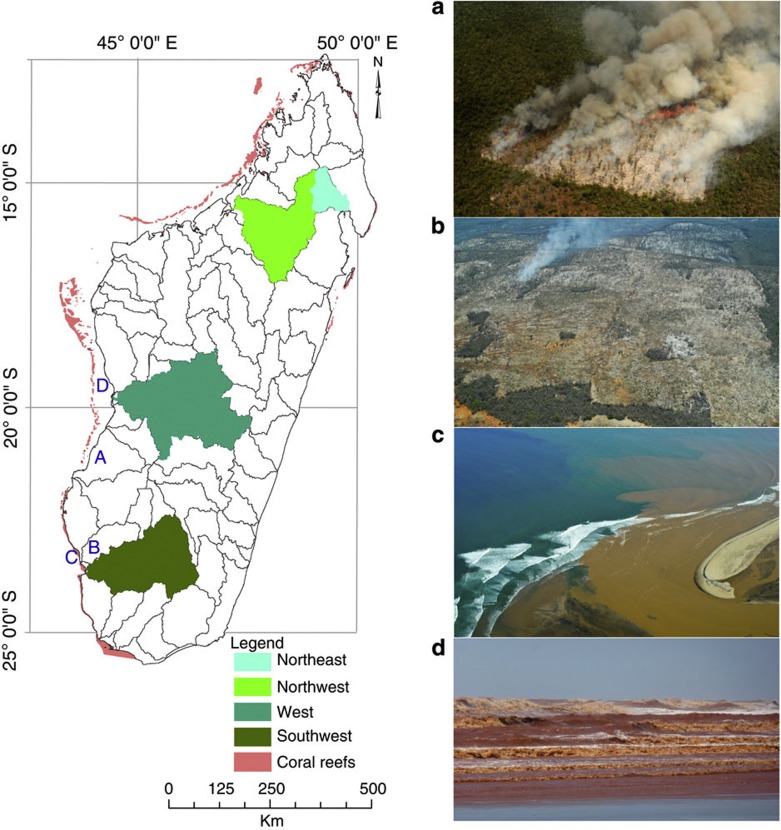
Map of Madagascar showing coral reef areas and the four studied watersheds. Images showing (**a** and **b**) deforestation (images courtesy of X. Vincke) and (**c** and **d**) reef sedimentation (panel **c** image courtesy of X. Vincke, panel **d** image courtesy of O. Raynaud). Images are shown with the corresponding letters on the map indicating approximate location of image capture.

**Figure 2:**
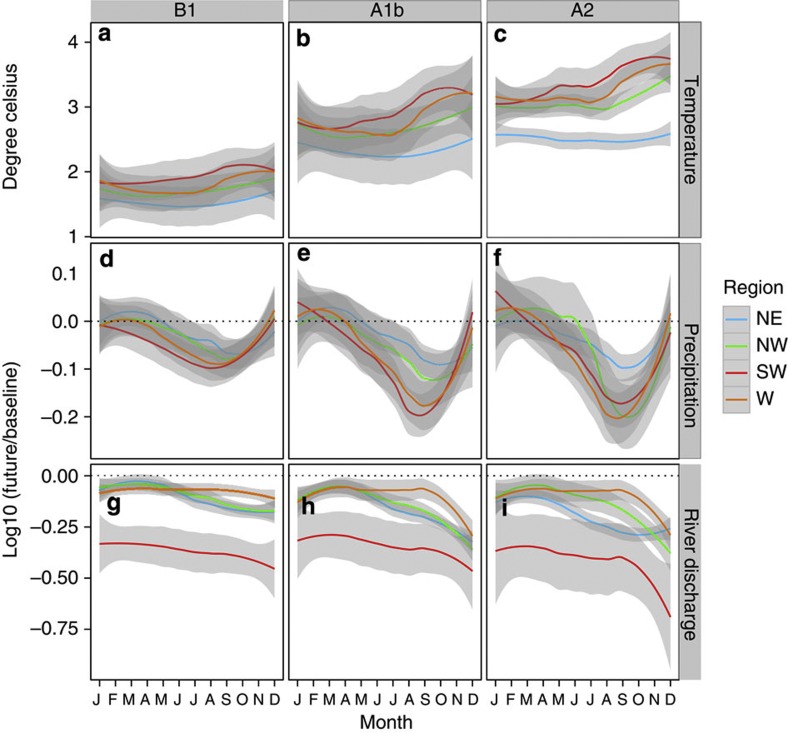
Predicted seasonal changes and simulated watershed discharge. (**a**–**c**) Predicted seasonal changes in temperature and (**d**–**f**) proportional changes in precipitation, and (**g**–**i**) simulated watershed discharge for the period 2070–2090 relative to the present day (1975–2005). A smooth line with 95% confidence interval (gray bands) representing variability in projections from different GCMs is fitted using Locally Weighted Scatterplot Smoothing (LOESS). Projections are presented by SRES scenarios (columns) and by region (colors).

**Figure 3:**
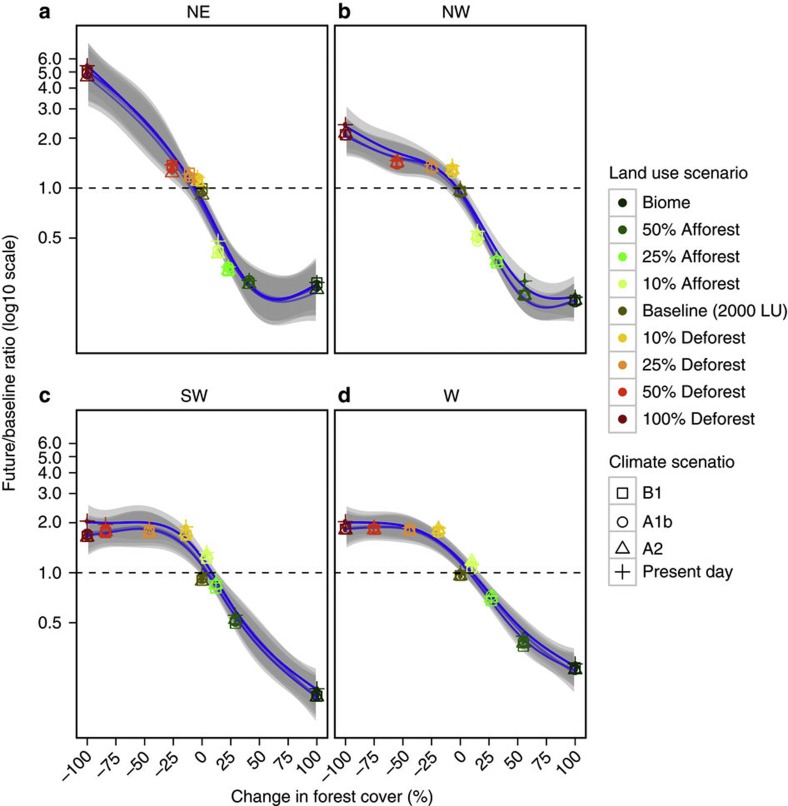
Predicted changes in sediment yield as a function of forest cover change. The panels are divided by region: (**a**) North-east, (**b**) North-west, (**c**) South-west, and (**d**) West. The mean predicted changes in sediment yields for each climate scenario (shapes) are presented for each region for the period 2070–2090 relative to the present day (1975–2005), against varying forest cover changes based on the different LULCC scenarios (color scale). A generalized linear model (GAM) smoother line for each climate scenario and the associated 95% confidence bands are displayed as blue lines and gray shades respectively.

The STREAM model is calibrated^6^ using temperature and precipitation monthly time series 55-km spatial resolution data (CRU version 3.1) (ref. 41). To process the future climate data for the hydrological model’s input, first we extracted 31-year baseline period (1975–2005) from the present-day climate data and computed averages for each month (that is, monthly climatology)^6^. Second, future precipitation and temperature data based on six GCM’s with three SRES scenarios represented (that is, A1b, A2 and B1, corresponding to 550, 700 and 850 p.p.m. atmospheric carbon dioxide in 2100, respectively) were obtained from World Climate Research Programme (WCRP) Coupled Model Inter-comparison Project (CMIP3) multi-model data set’s archives ( http://www-pcmdi.llnl.gov/projects/cmip/index.php). Finally, we applied a change-over-or time or delta-method^42^ to downscale the GCM projections to the present-day spatial resolution. In this method, from each of the individual time series (2000–2100), we selected a future 31-year period (2065–2095) from which we calculated 31-year running averages for each month, for grid cells intersecting the four Malagasy watersheds adjacent important coral reef areas (that is, north-east, north-west, west and south-west) (Fig. 1). Using the temperature baseline and future monthly climatologies, we computed anomalies or change maps for each month in the time series, as the absolute difference between future and present-day values. Similarly, we expressed precipitation as fractional changes in monthly mean precipitation, by dividing the future monthly climatology by the baseline monthly climatology. These changes in climate and precipitation were then applied to the present-day climate time series, to represent future climate at a relatively higher resolution.

### Land-use scenarios

To investigate the relative influence of forest cover on hydrology, we constructed nine LULCC scenarios as follows: (i) present-day land use, based on a land-use map for year 2000, which was developed from multi-temporal 1998–2001 satellite data^4^; (ii) completely forested or undisturbed, represented by the vegetation bio-climate map^24^; (iii–viii) incremental deforestation and afforestation targets (that is, 10, 25 and 50%), based on land-use change simulation, and (ix) completely deforested. The present-day land-use map is a combination of a vegetation distribution map distinguishing forested and non-forested areas^4^, and the Africover map for 2006 from the European Space Agency to fill in the non-forest cells. To simulate land-use change, we defined spatially explicit alternative futures that may result from future land management policies and socioeconomic drivers. Using present-day land use as the baseline, land-use changes for deforestation and afforestation targets at country level were simulated based on the slope and distance to roads.

For the afforestation and deforestation of cells, suitability for each scenario was computed for each cell, based on the slope and proximity to roads. For the 10% deforestation scenario, 10% of the forested cells with the highest suitability for deforestation (low slope and short distance to roads) in the whole of Madagascar were deforested. Note that this means that deforestation percentages in the four catchments considered in this study are therefore not necessarily the same as Madagascar as a whole. For afforestation, the reverse has been done. The 10% (or 25 or 50%, depending on the scenario) of the non-forest cells with the highest suitability to be forest (that is, high slope; far from roads) were transformed into forest cells. Again, as the 10% afforestation scenario refers to 10% in the whole of Madagascar, percentages in the individual catchments may differ. STREAM utilizes crop factors maps (that is, coefficients of evapotranspiration), for determining potential and actual evapotranspiration^35^. Crop factors based on land-cover characteristics are therefore derived using land-cover information. The land-cover maps were re-classified to the crop factor maps for use in STREAM, using a weighted average crop factor of non-forest use for the deforested cells.

### Rainfall erosivity

Rainfall erosivity is a measure for the erosive force of rainfall, and is a required input of RUSLE-based models including N-SPECT. Often, the large-scale mapping in data-poor regions is based on interpolation of erosivity values derived from rain-gauge data^38^. A few indices have been used to estimate erosivity, including the R-factor^43^, the Fournier Index (FI) and the modified Fournier Index (MFI)^38^. MFI when used with the monthly satellite-based precipitation was found to provide good spatial estimates of annual erosivity^44^. Annual spatial maps of MFI were computed for 1961–2006 (ref. 6)^6^.

### Soil data

Soil data was downloaded from Version 1.1 of the Harmonized soil database of the world (FAO/IIASA/ISRIC/ISSCAS/JRC 2009). STREAM utilizes the water-holding capacity of the soil as one of the input variables. The soil database was used in a reclassification procedure to develop the water-holding capacity map for input in STREAM^35^. N-SPECT utilizes the two variables derived from soils data: (i) hydrologic soil group (HSG’s)—soils are classified into four hydrologic soil group (A, B, C and D) to indicate the minimum rate of infiltration obtained for bare soil after prolonged wetting^45^. Using the soil types in the soil database, a soil hydrologic group map for Madagascar was derived using reclassification procedure; (ii) soil erodibility factor (K-Factor) represents soil’s susceptibility to erosion by rainstorms. It is an integrated average parameter based on several different erosion and hydrologic processes. K-factor is expressed as a function of sand, silt, clay and organic carbon concentration, which were derived using reclassification procedures using the soil database. A low K-factor (~0.05–0.2) indicates a high resistance to erosion and a high K-factor (~≥0.4) indicates easily eroded soil. K was computed as described in ref. 6.

### Elevation data

STREAM utilizes the flow direction map and the digital elevation model (DEM) maps as inputs. Hydrologically corrected DEM at 500 m resolution maps were downloaded from hydroSHEDS’s website^46^. N-SPECT utilizes DEM as an input factor where slope steepness (*S*) and slope length (*L*) that are derived from DEM are RUSLE parameters that adjusts erosion rates based on topography, assigning higher rates to longer or steeper slopes and lower rates to shorter or flatter ones^45^. N-SPECT also utilizes the DEM to delineate watersheds.

### Model calibration and runs

STREAM model was developed using present-day climate and land-use for the entire island^6^. The STREAM model was programmed to output the average monthly river flow in m^3^ s^-1^ for selected locations, which included outlets points of rivers, which drain each of the four regions (Fig.1). Observed data for monthly average river flows were taken from the RivDIS database (available at http://daac.ornl.gov/RIVDIS/rivdis.shtml)^42^. The period of record varies widely from station to station. We selected the station with the longest time series, Bevoay on River Mangoky (Global Runoff Data Centre Station Number 00059), and used it to calibrate the STREAM model^6^. The performance of the model was tested at every stage using the Nash and Sutcliffe model efficiency coefficient (NSE)^43^, and by taking the ratio of modeled to observed values. To simulate river discharge under present day and future climate, and under the LULCC scenarios, the respective climate and land-use data were substituted in the calibrated STREAM model^6^.

N-SPECT tool was run for each year from 1975–2006, with annual MFI, precipitation, land cover (crop factors), DEM and soil as the input variables. This provided with among other outputs, annual sediment yield per unit area in metric tonnes per year. To estimate sediment under GCM scenarios, relationships between present-day river flow (STREAM output) and present-day sediment load were established using bivariate regressions. River flow was summarized for each year (mean, maximum and minimum) and regressions performed between each of these statistics and present-day sediment output. Annual maximum average river flow was found to have a better fit with annual sediment yield than with annual averages and annual minimum. The relationships found were then applied to estimate corresponding future sediment.

## Author contributions

J.Z, J.M., H.dM. and J.V. designed the study. J.M., H.dM., J.S.M. and J.V. generated model outputs and conceived data interpretation. J.S.M. and T.M. contributed to design of land-use scenarios and choice of key study areas. TRM was supported by the John D. and Catherine T. MacArthur Foundation. All co-authors contributed to manuscript writing, performed model interpretations and discussions of the model scenario implications.

## Additional information

**How to cite this article:** Maina, J. *et al.* Human deforestation outweighs future climate change impacts of sedimentation on coral reefs. *Nat. Commun.* 4:1986 doi: 10.1038/ncomms2986 (2013).

## Figures and Tables

**Table 1:** Mean sediment change expressed as fractional change relative to the current estimates based on the 2000 land-use and present-day climate.

**LULCC**	**Climate scenario**	**NE**	**NW**	**SW**	**W**
Biome	B1	0.27	0.21	0.18	0.27
	A1B	0.26	0.21	0.19	0.27
	A2	0.25	0.21	0.19	0.27
	Present day	0.27	0.22	0.20	0.28

50% Afforest	B1	0.26	0.23	0.51	0.36
	A1B	0.27	0.23	0.53	0.39
	A2	0.26	0.22	0.53	0.37
	Present day	0.28	0.28	0.55	0.41

25% Afforest	B1	0.32	0.36	0.83	0.68
	A1B	0.33	0.37	0.86	0.73
	A2	0.32	0.35	0.87	0.71
	Present day	0.34	0.38	0.90	0.72

10% Afforest	B1	0.41	0.51	1.25	1.08
	A1B	0.41	0.49	1.23	1.07
	A2	0.43	0.54	1.32	1.17
	Present day	0.48	0.55	1.32	1.13

Baseline (2000 land use)	B1	0.98	0.97	0.92	0.96
	A1B	0.95	0.93	0.93	0.97
	A2	0.91	0.94	0.93	0.97
	Present day	3.69	4.49	5.09	3.53

10% Deforest	B1	1.10	1.30	1.73	1.77
	A1B	1.12	1.26	1.72	1.77
	A2	1.10	1.26	1.78	1.81
	Present day	1.16	1.36	1.89	1.86

25% Deforest	B1	1.22	1.35	1.76	1.80
	A1B	1.17	1.30	1.79	1.81
	A2	1.14	1.31	1.79	1.82
	Present day	1.21	1.39	1.91	1.87

50% Deforest	B1	1.36	1.42	1.81	1.83
	A1B	1.32	1.37	1.84	1.84
	A2	1.27	1.38	1.83	1.84
	Present day	1.38	1.47	1.95	1.88

100% Deforest	B1	5.04	2.14	1.69	1.83
	A1B	4.91	2.05	1.71	1.84
	A2	4.68	2.07	1.71	1.85
	Present day	5.45	2.41	2.05	2.03

The s.d. associated with the means ranges between 0.00–0.07. Sediment load fractional changes for the current estimates (that is, 2000 land use and present-day climate) are relative to the natural conditions, that is, the bio-climate map or biome.
